# Adolescent and Young Adult versus Older Adult Philadelphia-Negative Myeloproliferative Neoplasms: A Single-Center Experience from the MENA Region

**DOI:** 10.2147/JBM.S612879

**Published:** 2026-08-10

**Authors:** Shehab F Mohamed, Awni Alshurafa, Dina Sameh Soliman, Abdulrahman F Al-Mashdali, Mohammed Abdulgayoom, Anas M Babiker, Mahmoud Osman, Abdalla Fadul, Elmustafa Abdalla, Ahmed Abdelghafar Alsayed, Noor A Aweer, Manar E Abdel-Rahman, Omar Mohammad Ismail, Anil Yousaf, Mohamed Yassin

**Affiliations:** 1 Hematology Department, National Center for Cancer Care and Research (NCCCR), Hamad Medical Corporation, Doha, Qatar; 2 Department of Laboratory Medicine and Pathology, Hamad Medical Corporation, Doha, Qatar; 3 Clinical Pathology Department, National Cancer Institute, Cairo University, Cairo, Egypt; 4 Hamad Medical Corporation (HMC), Doha, Qatar; 5 Endocrinology Department, Hamad Medical Corporation, Doha, Qatar; 6 College of Medicine, Qatar University, Doha, Qatar; 7 Department of Public Health, College of Health Sciences, Qatar University, Doha, Qatar

**Keywords:** philadelphia-negative myeloproliferative neoplasms, adolescent and young adult, essential thrombocythaemia, polycythaemia vera, myelofibrosis, thrombosis

## Abstract

**Background:**

Philadelphia-negative myeloproliferative neoplasms (MPNs), including essential thrombocythaemia (ET), polycythaemia vera (PV), and myelofibrosis (MF), usually affect older adults, but a subset occurs in adolescents and young adults (AYA). Regional age-stratified data remain limited.

**Methods:**

We retrospectively studied Philadelphia-negative MPN patients managed at the National Center for Cancer Care and Research from 2014 to 2024. Patients were classified as AYA (15–39 years) or older adults (≥40 years). Clinical, laboratory, molecular, thrombotic, bleeding, transformation, and survival outcomes were analyzed by subtype. Risk categories followed institutional disease-specific practice; analyses were descriptive and unadjusted.

**Results:**

The cohort included 220 patients: 79 ET, 47 MF, and 94 PV; 76 (34.5%) were AYA. Low-risk disease predominated in AYA ET (p<0.001) and PV (72.4% vs 35.4%; p=0.002), partly reflecting age-based risk definitions. In ET (molecular n=30 vs 49), *CALR* was 16/30 (53.3%) versus 19/49 (38.8%) and *JAK2* was 6/30 (20.0%) versus 21/49 (42.9%); *MPL*, triple-negative, and unavailable categories were 2, 4, and 2 versus 2, 6, and 1, respectively. In MF (n=11 vs 36), *JAK2* was 4 (36.4%) versus 18 (50.0%), *CALR* 4 (36.4%) versus 10 (27.8%), *MPL* 0 versus 2 (5.6%), triple-negative 1 (9.1%) versus 3 (8.3%), and unavailable data 2 (18.2%) versus 3 (8.3%). In PV (n=29 vs 65), *JAK2* V617F was 28 (96.6%) versus 63 (96.9%) and *JAK2* exon 12 was 1 (3.4%) versus 2 (3.1%). Overall survival did not differ by age.

**Conclusion:**

AYA patients with Philadelphia-negative MPNs demonstrated age-related differences in risk classification, molecular profile, and thrombotic phenotype, while overall survival was comparable to older adults. Larger prospective multicenter studies are warranted.

## Plain Language Summary

We reviewed people with Philadelphia-negative myeloproliferative neoplasms treated at a single cancer center in Doha, Qatar. We compared younger patients aged 15-39 years with adults aged 40 years or above. Younger patients made up about one-third of the clinical cohort. In essential thrombocythaemia and polycythaemia vera, younger patients were more often placed in lower-risk groups, partly because standard risk systems include age and previous thrombosis. Molecular review showed *CALR* as the most frequent molecular category in essential thrombocythaemia, with relatively more *CALR*-positive and fewer *JAK2*-positive cases among AYA molecular records than older-adult molecular records. In myelofibrosis, *JAK2* was the most frequent category overall; *JAK2* and *CALR* were equally represented in AYA patients, while *JAK2* was most frequent in older adults. In polycythaemia vera, all patients had available molecular data; most had *JAK2* V617F, and three had *JAK2* exon 12 mutations. Some age-related differences were seen in thrombosis type, but these were not statistically significant. Overall survival did not significantly differ between age groups during available follow-up. These findings are exploratory and show the need for larger Middle Eastern studies with standardized treatment, molecular, pregnancy, and quality-of-life data.

## Introduction

Classical Philadelphia-negative myeloproliferative neoplasms (MPNs) comprise essential thrombocythaemia (ET), polycythaemia vera (PV), and primary myelofibrosis (PMF/MF). These clonal myeloid neoplasms are characterized by chronic overproduction of mature blood cells, thrombohemorrhagic complications, symptom burden, and a risk of fibrotic or leukemic transformation.^1–3^ Although MPNs are traditionally diagnosed in later adult life, an increasingly recognized subset arises in adolescents and young adults (AYA), typically defined as 15–39 years of age.^4–6^

The AYA population with MPN is clinically important because prolonged disease duration, fertility and pregnancy issues, treatment sequencing, psychosocial burden, and late thrombotic or transformation risks may differ from those of older adults.^4^,^7–9^ Potential mechanisms underlying age-associated differences include lower cumulative exposure to cardiovascular risk factors, differences in driver mutation distribution and allele burden, and differences in clonal evolution over time. Prior studies suggest that younger patients may demonstrate distinct biologic patterns, including lower *JAK2* allele burden, relative enrichment of *CALR* mutations in ET, and a predilection for venous or splanchnic thrombosis rather than arterial events.^4–12^ At the same time, overall survival in younger patients is often favorable, particularly in ET and PV, although disease progression remains a long-term concern.^4^,^6^,^13^,^14^

Most published AYA data come from North American and European cohorts, while data from the Middle East are scarce. Regional demographic structure, referral patterns, and ethnic diversity may influence the age distribution and phenotypic spectrum of MPNs. We therefore conducted a retrospective single-center study at the National Center for Cancer Care and Research (NCCCR), Doha, Qatar, to characterize AYA patients with Philadelphia-negative MPNs and compare them with older adults across ET, MF, and PV. We hypothesized that AYA patients would demonstrate differences in risk classification, driver-mutation distribution, and thrombotic phenotype compared with older adults, while recognizing that some risk-category differences are expected because age is embedded within standard prognostic models.

## Methods

Study design and population: This retrospective single-center cohort included consecutive patients with ET, PV, or MF diagnosed and/or followed at the National Center for Cancer Care and Research (NCCCR), Doha, Qatar, from 2014 to 2024. Patients were stratified into AYA (15–39 years) and older adults (≥40 years).

Data collection and molecular review: Demographic, baseline laboratory, molecular, cytogenetic, marrow fibrosis, splenomegaly, bleeding, thrombosis, transformation, and survival data were extracted from institutional records. Molecular data were re-reviewed for ET, MF, and PV, and final age-stratified molecular results are reported in the tables.

Risk stratification and treatment exposure: ET risk grouping used an adapted revised IPSET-thrombosis approach, PV risk grouping used conventional criteria (high risk if age >60 years and/or prior thrombosis), and MF categories used documented IPSS/DIPSS-derived risk groups. Cytoreductive therapy, JAK inhibitor exposure, aspirin, anticoagulation, treatment duration, and treatment sequencing were not consistently extractable and were not incorporated into comparative or survival analyses.

Outcomes and statistics: Outcomes included baseline features, documented thrombosis and bleeding, transformation, progression-free survival where available, and overall survival. Observation time extended from diagnosis to last encounter or death; non-fatal events are reported as cumulative documented events rather than person-year incidence rates because exact event dates were not uniformly available. Categorical variables were compared with chi-square or Fisher exact tests, continuous variables are presented as median (IQR), and survival was analyzed by Kaplan-Meier/log-rank methods. Analyses were descriptive and unadjusted; multivariable models were not performed because event numbers were low and key covariates were incomplete.

Ethics: Institutional Review Board approval was obtained prior to study conduct.

## Results

A total of 220 patients with Philadelphia-negative MPNs were included in the age-stratified clinical and outcome analyses, comprising 79 patients with essential thrombocythaemia (ET), 47 with myelofibrosis (MF), and 94 with polycythaemia vera (PV). Overall, 76 patients (34.5%) were classified as adolescent and young adults (AYA; 15–39 years), including 36/79 (45.6%) with ET, 11/47 (23.4%) with MF, and 29/94 (30.9%) with PV.

### Essential Thrombocythaemia

Among the 79 patients with ET, AYA patients were predominantly female (61.1%), whereas older adults showed a male predominance (58.1%). Median age at diagnosis was 28.5 years (IQR 25.5–33.0) in the AYA group and 54.0 years (IQR 47.0–62.0) in older adults. Asian nationality represented the largest subgroup in both age categories, accounting for 50.0% of AYA and 74.4% of older ET cases.

The most apparent age-based difference in ET was risk distribution. Low-risk disease accounted for 80.0% of evaluable AYA cases compared with none of the older adults, while intermediate- and high-risk disease predominated among patients aged 40 years or older. This result should be interpreted in the context of the risk model because age and prior thrombosis are central components of ET risk classification and can produce expected differences between age-defined groups. Among 79 patients with ET, *CALR*-positive disease was the most frequent category (35/79, 44.3%), followed by *JAK2*-positive disease (27/79, 34.2%), triple-negative disease (10/79, 12.7%), *MPL*-positive disease (4/79, 5.1%), and unavailable molecular data (3/79, 3.8%). In the age-stratified molecular summary, *CALR*-positive disease was more frequent among AYA molecular records (16/30, 53.3%) than older-adult molecular records (19/49, 38.8%), whereas *JAK2*-positive disease was less frequent among AYA molecular records (6/30, 20.0%) than older-adult molecular records (21/49, 42.9%). Because the molecular-profile denominators differed from the clinical age-group denominators, these age-specific molecular comparisons should be interpreted descriptively. Median leukocyte count, haemoglobin, platelet count, and absolute lymphocyte count were otherwise broadly similar between the two age groups on descriptive analysis. Detailed baseline ET characteristics are summarized in Table 1.

**Table 1:** Baseline Demographic and Clinical Characteristics of Essential Thrombocythaemia by Age Group

Variable	Age 15–39 Years(n=36)	Age ≥ 40 Years(n=43)	Total(n=79)
**Sex, n (%)**			
Male	14 (38.9)	25 (58.1)	39 (49.4)
Female	22 (61.1)	18 (41.9)	40 (50.6)
Age at diagnosis, median [IQR]	28.5 [25.5–33.0]	54.0 [47.0–62.0]	41.0 [29.0–55.0]
**Nationality, n (%)**			
Asian	18 (50.0)	32 (74.4)	50 (63.3)
African	9 (25.0)	7 (16.3)	16 (20.3)
White	9 (25.0)	4 (9.3)	13 (16.5)
**Risk category (n=72), n (%)**			
Low*	28 (80.0)	0 (0.0)	28 (39.4)
Intermediate	1 (2.9)	15 (41.7)	16 (22.5)
High	6 (17.1)	21 (58.3)	27 (38.0)
**Driver mutation category (n=79), n (%)†**			
*JAK2*-positive	6 (20.0)	21 (42.9)	27 (34.2)
*CALR*-positive	16 (53.3)	19 (38.8)	35 (44.3)
*MPL*-positive	2 (6.7)	2 (4.1)	4 (5.1)
Triple-negative	4 (13.3)	6 (12.2)	10 (12.7)
NA (no available data)	2 (6.7)	1 (2.0)	3 (3.8)
WBC count at diagnosis (x10^9/L), median [IQR]	9.2 [7.8–11.1]	8.2 [7.0–10.9]	8.8 [7.2–10.9]
Hemoglobin (g/dL), median [IQR]	13.3 [12.4–14.7]	13.3 [12.4–14.7]	13.3 [12.4–14.7]
Platelet count (x10^9/L), median [IQR]	735.0 [300.0–895.0]	747.5 [537.0–898.0]	747.0 [499.0–895.0]
Absolute lymphocyte count, median [IQR]	2.5 [2.0–2.9]	2.1 [1.4–2.6]	2.3 [1.6–2.8]
**Splenomegaly, n (%)**			
No	14 (38.9)	16 (37.2)	30 (38.0)
Yes (>12 cm)	22 (61.1)	27 (62.8)	49 (62.0)

**Notes**: *For ET, the low category combines very-low and low categories from the adapted revised IPSET-thrombosis framework. The molecular profile includes 79 reviewed molecular records: *CALR*-positive 35/79 (44.3%), *JAK2*-positive 27/79 (34.2%), *MPL*-positive 4/79 (5.1%), triple-negative 10/79 (12.7%), and unavailable data 3/79 (3.8%).

**Figure 1:**
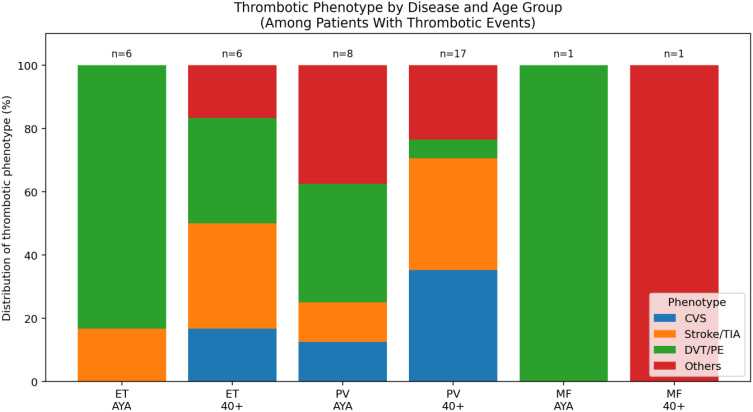
Thrombotic phenotype by disease subtype and age group among patients with documented thrombotic events. In ET and PV, AYA patients showed a relatively greater proportion of venous thrombotic events (DVT/PE), whereas older adults showed a higher proportion of cerebrovascular and other arterial events. Bars are restricted to patients with thrombotic events; n above each bar indicates the number of thrombotic events in that subgroup. Because absolute event numbers are small, the figure should be interpreted descriptively.

Bleeding and thrombosis were uncommon in ET overall. Minor bleeding occurred in one AYA patient and major bleeding in one older adult. Most patients in both age groups had no thrombotic event; however, venous thrombosis (DVT/PE) was numerically more frequent in AYA ET patients (13.9% vs 4.7%), while cerebrovascular and other arterial events were observed mainly in older adults. These event comparisons are descriptive because the absolute event numbers were small and follow-up duration was heterogeneous. Splenomegaly was common in both groups, affecting 61.1% of AYA and 62.8% of older adults. The distribution of thrombotic phenotypes across disease subtypes and age groups is illustrated in Figure 1.

### Myelofibrosis

Among the 47 patients with MF, 11 were AYA and 36 were older adults. Sex distribution was similar between groups, and the median age at diagnosis was 35.0 years in AYA patients compared with 54.0 years in older adults. Asian nationality was the most common background in both age strata. In contrast to ET and PV, MF showed a broadly comparable baseline clinical phenotype across age groups.

Risk category, cytogenetic findings, marrow fibrosis grade, and splenomegaly did not show major age-related differences on descriptive review. Risk distribution in MF was low risk in 5/11 (45.5%) AYA versus 12/36 (33.3%) older adults, intermediate risk in 3/11 (27.3%) versus 20/36 (55.6%), high risk in 3/11 (27.3%) versus 3/36 (8.3%), with one older patient missing risk data. On molecular review, *JAK2*-positive disease was documented in 4/11 (36.4%) AYA versus 18/36 (50.0%) older adults, *CALR*-positive disease in 4/11 (36.4%) versus 10/36 (27.8%), *MPL*-positive disease in 0 versus 2/36 (5.6%), triple-negative disease in 1/11 (9.1%) versus 3/36 (8.3%), and unavailable molecular data in 2/11 (18.2%) versus 3/36 (8.3%), respectively. Overall, the MF molecular profile included *JAK2*-positive disease in 22/47 (46.8%), *CALR*-positive disease in 14/47 (29.8%), *MPL*-positive disease in 2/47 (4.3%), triple-negative disease in 4/47 (8.5%), and unavailable data in 5/47 (10.6%). Grade 2–3 marrow fibrosis was present in 80.0% of AYA and 60.0% of older adults with available biopsy data. Splenomegaly was highly prevalent overall, affecting 90.9% of AYA and 77.8% of older adults. Transformation was documented only in older adults (8.3%), whereas none of the AYA MF patients transformed during follow-up. Bleeding and thrombotic complications were infrequent in both groups, with only isolated events recorded. Detailed baseline MF characteristics and molecular distribution are summarized in Table 2.

**Table 2:** Baseline Demographic and Clinical Characteristics of Myelofibrosis by Age Group

Variable	Age 15–39 Years (n=11)	Age ≥40 Years (n=36)	Total (n=47)
**Sex, n (%)**			
Male	6 (54.5)	21 (58.3)	27 (57.4)
Female	5 (45.5)	15 (41.7)	20 (42.6)
Age at diagnosis, median [IQR]	35.0 [27.0–38.0]	54.0 [45.0–61.0]	49.5 [39.0–56.0]
**Nationality, n (%)**			
Asian	6 (54.5)	20 (55.6)	26 (55.3)
African	2 (18.2)	10 (27.8)	12 (25.5)
White	3 (27.3)	6 (16.7)	9 (19.1)
**Risk category, n (%)**			
Low	5 (45.5)	12 (33.3)	17 (36.2)
Intermediate*	3 (27.3)	20 (55.6)	23 (48.9)
High	3 (27.3)	3 (8.3)	6 (12.8)
Missing	0 (0.0)	1 (2.8)	1 (2.1)
**Driver mutation category, n (%)**			
*JAK2*-positive	4 (36.4)	18 (50.0)	22 (46.8)
*CALR*-positive	4 (36.4)	10 (27.8)	14 (29.8)
*MPL*-positive	0 (0.0)	2 (5.6)	2 (4.3)
Triple-negative	1 (9.1)	3 (8.3)	4 (8.5)
NA (no available data)	2 (18.2)	3 (8.3)	5 (10.6)
**Cytogenetics, n (%)**			
Normal (diploid)	11 (100.0)	33 (91.7)	44 (93.6)
Abnormal	0 (0.0)	3 (8.3)	3 (6.4)
WBC count at diagnosis (x10^9/L), median [IQR]	9.0 [6.4–11.9]	8.7 [5.9–13.2]	8.8 [6.4–12.3]
Hemoglobin (g/dL), median [IQR]	11.1 [8.1–14.0]	11.3 [9.6–13.2]	11.3 [9.4–13.4]
Platelet count (x10^9/L), median [IQR]	289.0 [232.0–921.0]	341.0 [195.0–712.0]	341.0 [198.0–763.0]
**Bone marrow fibrosis, n (%)**			
Grade 0-1	2 (20.0)	12 (40.0)	14 (35.0)
Grade 2-3	8 (80.0)	18 (60.0)	26 (65.0)
**Splenomegaly, n (%)**			
No	1 (9.1)	8 (22.2)	9 (19.1)
Yes (>12 cm)	10 (90.9)	28 (77.8)	38 (80.9)

**Notes**: *For MF, documented IPSS/DIPSS intermediate-1 and intermediate-2 categories were collapsed into a single intermediate category. The MF molecular profile includes all 47 patients; molecular row denominators are the same as the clinical age groups (11 AYA and 36 older adults).

### Polycythaemia Vera

In the PV cohort, 29 patients were AYA and 65 were older adults. AYA patients were more often male (75.9% vs 53.8%), and median age at diagnosis was 33.0 years (IQR 30.0–35.0) in AYA patients compared with 58.0 years (IQR 50.0–65.0) in older adults. Asian nationality predominated in both age groups, accounting for 72.4% of AYA and 83.1% of older adult patients.

Risk distribution differed in PV, with low-risk disease observed in 72.4% of AYA patients compared with 35.4% of older adults, while high-risk disease predominated among the older group (64.6%). As in ET, this risk distribution should not be interpreted as purely biologic because conventional PV risk grouping directly incorporates age >60 years and prior thrombosis. On molecular review, *JAK2* V617F was documented in 28/29 (96.6%) AYA and 63/65 (96.9%) older adults, while *JAK2* exon 12 mutations were documented in 1/29 (3.4%) AYA and 2/65 (3.1%) older adults. All 94 PV patients had available molecular results. Cytogenetic abnormalities were infrequent and observed mainly among older adults.

Median leukocyte count at diagnosis was numerically lower in AYA patients (9.2 x10^9/L) than in older adults (11.6 x10^9/L), whereas haemoglobin and platelet counts were broadly comparable. Splenomegaly was observed in 79.3% of AYA patients and 63.1% of older adults. No AYA PV patient experienced bleeding, while minor and major bleeding were documented only in older adults. Thrombotic events were absent in most cases, but venous thrombosis (DVT/PE) appeared relatively more common in AYA PV patients, while cerebrovascular events and stroke/TIA were more often recorded in older adults. These comparisons are descriptive and should be interpreted in light of small event numbers and variable follow-up. Detailed baseline PV characteristics are summarized in Table 3, while bleeding, thrombotic, and transformation outcomes across disease subtypes are consolidated in Table 4.

**Table 3:** Baseline Demographic and Clinical Characteristics of Polycythaemia Vera by Age Group

Variable	Age 15–39 Years (n=29)	Age ≥40 Years (n=65)	Total (n=94)
**Sex, n (%)**			
Male	22 (75.9)	35 (53.8)	57 (60.6)
Female	7 (24.1)	30 (46.2)	37 (39.4)
Age at diagnosis, median [IQR]	33.0 [30.0–35.0]	58.0 [50.0–65.0]	50.5 [36.0–62.0]
**Nationality, n (%)**			
Asian	21 (72.4)	54 (83.1)	75 (79.8)
African	7 (24.1)	7 (10.8)	14 (14.9)
White	1 (3.4)	3 (4.6)	4 (4.3)
Others	0 (0.0)	1 (1.5)	1 (1.1)
Risk category, n (%)*			
Low	21 (72.4)	23 (35.4)	44 (46.8)
High	8 (27.6)	42 (64.6)	50 (53.2)
**Molecular result** **			
*JAK2* V617F	28 (96.6)	63 (96.9)	91 (96.8)
*JAK2* exon 12	1 (3.4)	2 (3.1)	3 (3.2)
NA (no available data)	0 (0.0)	0 (0.0)	0 (0.0)
**Cytogenetics (n=80), n (%)**			
Normal (diploid)	25 (96.2)	50 (92.6)	75 (93.8)
Abnormal	1 (3.8)	4 (7.4)	5 (6.2)
WBC count at diagnosis (x10^9/L), median [IQR]	9.2 [7.3–11.1]	11.6 [8.2–15.2]	10.5 [7.5–14.6]
Hemoglobin (g/dL), median [IQR]	16.2 [14.4–16.9]	15.4 [13.8–16.6]	15.6 [13.9–16.8]
Hematocrit (%), median [IQR]	48.1 [44.5–51.4]	47.8 [42.8–53.5]	48.1 [43.3–53.0]
**Splenomegaly, n (%)**			
No	6 (20.7)	24 (36.9)	30 (31.9)
Yes (>12 cm)	23 (79.3)	41 (63.1)	64 (68.1)

**Notes**: *PV risk classification was based on conventional criteria: high risk if age >60 years and/or prior thrombosis; low risk if neither feature was present. **The molecular profile included 91 *JAK2* V617F cases and 3 *JAK2* exon 12 cases. Molecular row denominators are the same as the clinical age groups (29 AYA and 65 older adults).

**Table 4:** Bleeding, Thrombotic, and Transformation Outcomes by Subtype and Age Group

Disease / Outcome	Age 15–39 Years	Age ≥40 Years	Total
**ET outcomes**	36	43	79
**Severity of bleeding events, n (%)**			
No event	35 (97.2)	42 (97.7)	77 (97.5)
Minor event	1 (2.8)	0 (0.0)	1 (1.3)
Major event	0 (0.0)	1 (2.3)	1 (1.3)
**Thrombotic event, n (%)**			
None	30 (83.3)	37 (86.0)	67 (84.8)
CVS	0 (0.0)	1 (2.3)	1 (1.3)
Stroke/TIA	1 (2.8)	2 (4.7)	3 (3.8)
DVT/PE	5 (13.9)	2 (4.7)	7 (8.9)
Others	0 (0.0)	1 (2.3)	1 (1.3)
**MF outcomes**	11	36	47
**Transformation, n (%)**			
No	11 (100.0)	33 (91.7)	44 (93.6)
Yes	0 (0.0)	3 (8.3)	3 (6.4)
**Severity of bleeding events, n (%)**			
No event	11 (100.0)	34 (94.4)	45 (95.7)
Minor event	0 (0.0)	1 (2.8)	1 (2.1)
Major event	0 (0.0)	1 (2.8)	1 (2.1)
**Thrombotic event, n (%)**			
None	10 (90.9)	35 (97.2)	45 (95.7)
DVT/PE	1 (9.1)	0 (0.0)	1 (2.1)
Others	0 (0.0)	1 (2.8)	1 (2.1)
**PV outcomes**	29	65	94
**Severity of bleeding events, n (%)**			
No event	29 (100.0)	58 (89.2)	87 (92.6)
Minor event	0 (0.0)	4 (6.2)	4 (4.3)
Major event	0 (0.0)	3 (4.6)	3 (3.2)
**Thrombotic event, n (%)**			
None	21 (72.4)	48 (73.8)	69 (73.4)
CVS	1 (3.4)	6 (9.2)	7 (7.4)
Stroke/TIA	1 (3.4)	6 (9.2)	7 (7.4)
DVT/PE	3 (10.3)	1 (1.5)	4 (4.3)
Others	3 (10.3)	4 (6.2)	7 (7.4)

**Notes**: Cumulative documented events are reported during available follow-up; exact time-to-event dates for non-fatal events were not uniformly available.

**Abbreviations**: CVS, cardiovascular system; DVT/PE, deep vein thrombosis/pulmonary embolism; TIA, transient ischemic attack.

### Overall Survival

**Figure 2:**
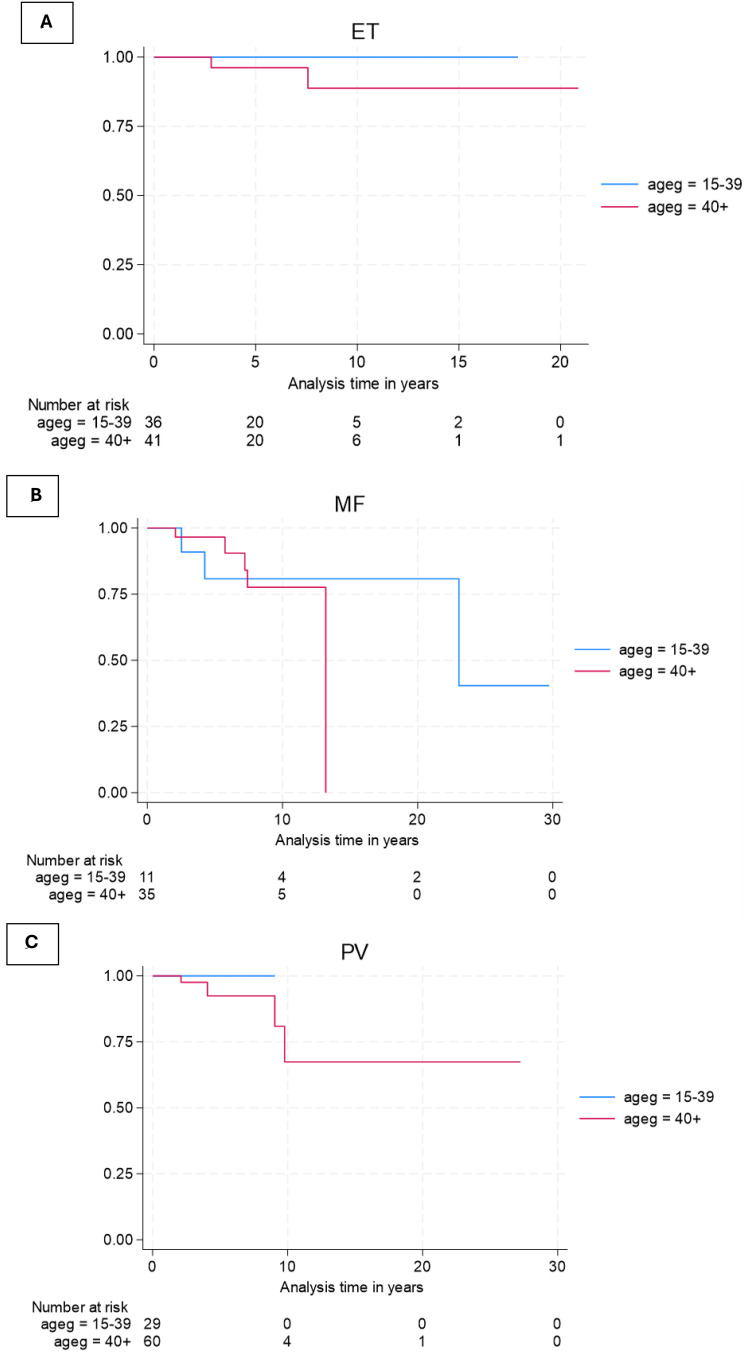
(**A**) Kaplan-Meier overall survival curves for essential thrombocythaemia (ET) stratified by age group. No statistically significant difference in overall survival was observed between AYA and older adults (log-rank p=0.1496). Survival analyses were unadjusted for treatment exposure and cardiovascular risk factors. (**B**) Kaplan-Meier overall survival curves for myelofibrosis (MF) stratified by age group. No statistically significant difference in overall survival was observed between AYA and older adults (log-rank p=0.650). Survival analyses were unadjusted for treatment exposure and cardiovascular risk factors. (**C**) Kaplan-Meier overall survival curves for polycythaemia vera (PV) stratified by age group. No statistically significant difference in overall survival was observed between AYA and older adults (log-rank p=0.336). Survival analyses were unadjusted for treatment exposure and cardiovascular risk factors.

Kaplan-Meier analyses did not demonstrate statistically significant overall survival differences between AYA and older adults in any disease subtype. The log-rank p values were 0.1496 for ET, 0.650 for MF, and 0.336 for PV. The corresponding Kaplan-Meier overall survival curves are shown in Figure 2A–C. These analyses were unadjusted and were not stratified by cytoreductive therapy, JAK inhibitor exposure, antiplatelet therapy, anticoagulation, or cardiovascular risk factors because these variables were not uniformly captured in the retrospective dataset.

**Figure 3:**
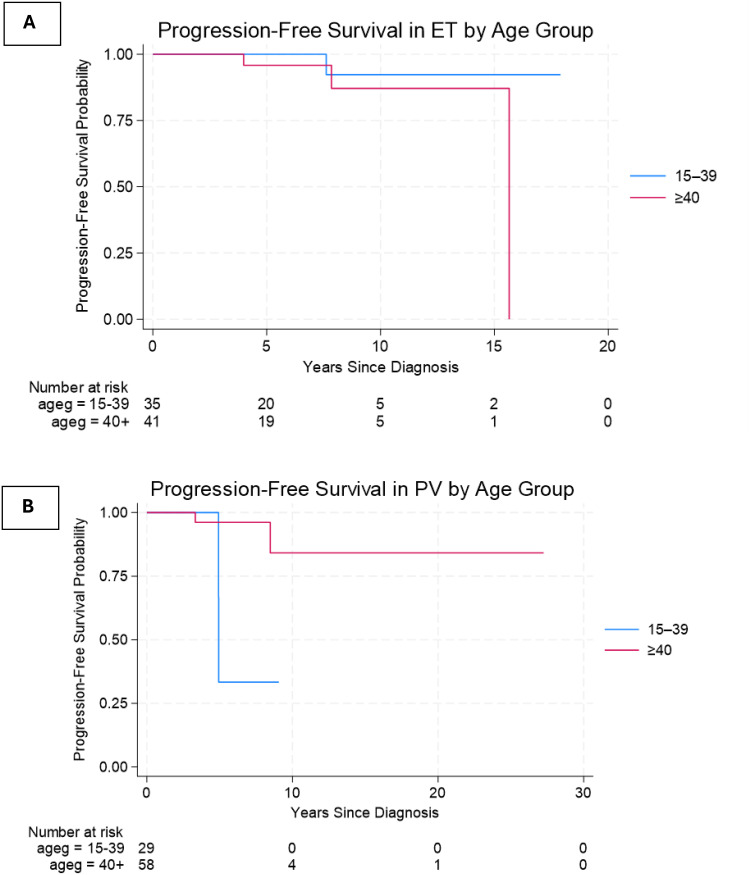
(**A**) Descriptive Kaplan-Meier curves of progression-free survival in patients with essential thrombocythaemia (ET), stratified by age group. These curves should be interpreted cautiously because progression events were few and treatment exposure was not incorporated into the analysis. (**B**) Descriptive Kaplan-Meier curves of progression-free survival in patients with polycythaemia vera (PV), stratified by age group. These curves should be interpreted cautiously because progression events were few and treatment exposure was not incorporated into the analysis.

These findings should be interpreted cautiously because the number of deaths was low in several subgroups, event rates were modest, and follow-up duration may still be insufficient to capture the full long-term trajectory of younger patients with chronic MPNs. Descriptive Kaplan-Meier progression-free survival curves for ET and PV are shown in Figure 3A and B; these curves should also be considered exploratory because of limited event counts and incomplete treatment-adjustment data.

## Discussion

**Figure 4:**
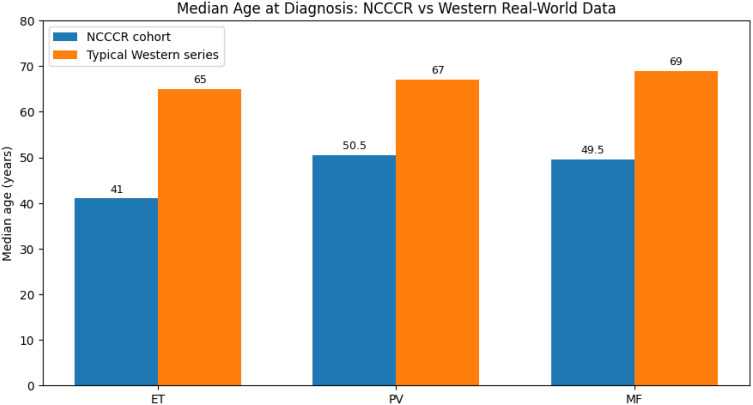
Median age at diagnosis in the NCCCR cohort versus representative Western real-world series across ET, PV, and MF. The NCCCR cohort showed younger median age at diagnosis across all three disease subtypes.

In the present NCCCR cohort, patients with Philadelphia-negative MPNs appeared younger across all three classical entities than would be expected from many real-world Western series. The median age at diagnosis in our cohort was 41.0 years for ET, 50.5 years for PV, and 49.5 years for MF, whereas classical MPNs are often described as diseases diagnosed in the seventh decade, with reported median ages of approximately 65 years for ET, 67 years for PV, and 69 years for PMF.^4^ This age shift likely reflects the demographic structure of Qatar, the expatriate-heavy working-age population served by our center, and referral patterns within a national tertiary cancer program. Importantly, it provides essential context for interpretation of our age-stratified findings: our AYA cohort is genuinely young, but the comparison group is also younger than the older populations typically represented in European and North American datasets. This age contrast relative to representative Western real-world series is illustrated in Figure 4.

This point may help explain why several clinicobiologic differences between AYA and older adults in our series were directionally similar to the literature but did not always reach large effect sizes or statistical significance. In our cohort, the median age of the older group was 54.0 years in ET, 54.0 years in MF, and 58.0 years in PV, which is older than AYA by definition but still younger than the populations often used to define older MPN cohorts in the United States and Europe, where many comparator groups are dominated by patients aged 60 years and above.^4^,^10^,^14^ As a result, our study may be capturing a comparison between young adults and middle-aged adults rather than between young adults and elderly patients, thereby attenuating age-related contrasts in survival, thrombosis burden, symptom biology, and treatment exposure.

This retrospective single-center study provides one of the few descriptions of AYA Philadelphia-negative MPNs from the Gulf region and demonstrates that age at diagnosis is associated with differences in disease phenotype, particularly in ET and PV. The most consistent signal in our cohort was a lower-risk distribution among AYA patients, most clearly observed in ET and PV. However, this finding should be interpreted cautiously and not overread as an independent biologic feature of AYA-MPN because age and prior thrombosis are incorporated into standard risk systems. Therefore, the observed risk distribution is partly expected from the structure of the scoring models, in addition to potentially reflecting a lower burden of age-related cardiovascular comorbidity in younger adults.^2^,^4^,^10^

Our ET findings align with the broader literature suggesting that younger patients often have a more indolent baseline phenotype, yet carry a long future exposure time for thrombosis, progression, and treatment-related decisions.^4^,^6^,^7^,^13^ The ET molecular profile showed *CALR*-positive disease as the most frequent category overall, followed by *JAK2*-positive disease, triple-negative disease, *MPL*-positive disease, and unavailable results. Age-stratified molecular counts suggested relative *CALR* enrichment among AYA molecular records and relatively higher *JAK2* positivity among older-adult molecular records; however, because molecular and clinical denominators differed, these age-specific molecular observations should remain descriptive. The overall pattern is directionally consistent with prior reports showing *CALR*-mutated ET to be common in younger or lower-risk ET populations and associated with distinct clinical behavior.^4^,^6^,^11^,^12^

In PV, the greater proportion of low-risk disease among AYA patients is consistent with prior age-stratified studies but is also strongly influenced by the age component of conventional PV risk classification.^4^,^10^ The molecular review showed *JAK2* positivity, including exon 12, in 27/28 (96.4%) patients <40 years and 66/66 (100.0%) patients ≥40 years with molecular data; overall, the PV molecular profile included 90 *JAK2* V617F cases, 3 *JAK2* exon 12 cases, and 1 case with unavailable molecular data. Although we did not detect statistically significant differences in thrombosis, the pattern in our data is notable: venous events appeared relatively more prominent in AYA patients, whereas cerebrovascular events were more often seen in older adults. This mirrors published AYA MPN cohorts in which younger patients demonstrate a relative excess of venous and splanchnic thrombosis, while arterial events are more typical of older adults.^4–10^

In contrast, MF showed fewer age-related clinical differences in our study. Molecular profiling showed that, *JAK2* and *CALR* were equally represented in AYA patients, whereas *JAK2*-positive disease predominated among older adults. These observations should be interpreted cautiously because the AYA MF subgroup included only 11 patients. The limited clinical differences likely reflect the smaller number of AYA MF patients rather than a true absence of biologic heterogeneity. Nonetheless, the descriptive observation that transformation occurred only in older adults and that splenomegaly was common in both age groups is clinically relevant. Published series of younger MF/PMF patients have suggested that although survival may be longer, meaningful morbidity persists and disease evolution remains possible over time.^4^,^6^,^14^

Our cohort also highlights the importance of geographic context. AYA patients comprised more than one-third of the overall cohort, which is higher than in many published Western series.^4^,^5^,^14^ This may reflect the younger population structure of Qatar, referral pathways to a national tertiary cancer center, or the multinational demographic composition of the local population. The marked predominance of Asian and African patients across several subgroups further underscores the value of reporting data from underrepresented regions.

The clinical implications of our findings are several. First, age-specific interpretation of risk in MPN remains essential, but low-risk classification in AYA should not be equated with trivial disease or interpreted without considering how risk models are constructed. Second, thrombotic phenotype may differ by age even when aggregate event rates are similar, supporting careful assessment for venous events in younger patients. Third, because AYA patients may live with their disease for decades, treatment selection should incorporate long-term goals including fertility preservation, cumulative toxicity, disease modification where appropriate, adherence, and quality of life.^4^,^7^,^8^,^15^

### Pregnancy Considerations

Pregnancy deserves explicit discussion in any AYA-focused MPN study, although pregnancy-specific outcomes, delivery outcomes, and obstetric complications were not systematically captured in this retrospective cohort. Therefore, the following discussion is contextual rather than a cohort-derived pregnancy analysis. In our center, this issue is particularly relevant because ET was the most common diagnosis among young women, and AYA ET patients showed a female predominance. Contemporary literature suggests that MPN pregnancies are uncommon but clinically important, with higher rates of miscarriage, preterm birth, and placental complications than in the general population.^16^,^17^ In a systematic review and meta-analysis of 1210 MPN pregnancies, the pooled live birth rate was 71.3%, and aspirin as well as interferon were associated with higher odds of live birth.^16^ Similarly, in ET-specific cohorts, prior pregnancy loss has emerged as an important predictor of subsequent complications, underscoring the need for individualized counselling before conception.^18^

At NCCCR, these data support a multidisciplinary approach involving both a hematologist familiar with MPN biology and a maternal-fetal medicine specialist for high-risk pregnancy care. To support this need, we have established a dedicated hematology-obstetric clinic jointly run by hematology and obstetric consultants. This clinic provides multidisciplinary assessment and coordinated decision-making regarding conception planning, antepartum management, thrombosis prevention, cytoreductive therapy when needed, and delivery planning for women with MPN who become pregnant. Low-dose aspirin is generally appropriate in the absence of a major bleeding tendency, and pegylated interferon remains the preferred cytoreductive option when cytoreduction is required during pregnancy.^16^,^17^ Post-partum thromboprophylaxis with low-molecular-weight heparin should be considered carefully given the recognized increase in post-partum venous thromboembolic risk.^17^ While mutation-specific counseling remains challenging, available data suggest that *JAK2*-positive disease may be associated with poorer pregnancy outcomes in some cohorts, whereas *CALR*-mutated ET may have a more favorable obstetric profile, although these associations remain inconsistent across studies.^18^,^19^ In our setting, the practical message is that reproductive counselling should begin early, ideally at diagnosis, because treatment planning, thrombosis prevention, and contraception choices are often intertwined in young women with MPN.

### Quality of Life and Psychosocial Implications

Quality of life was not formally captured in our retrospective dataset; nevertheless, the age distribution of our cohort makes this domain highly relevant. Landmark survey data have shown that symptom burden adversely affects daily function, work productivity, and overall quality of life even among patients with low-risk ET and PV.^20^,^21^ For AYA patients, the impact likely extends beyond missed work days to interrupted education, career development, family planning, and long-term financial stability. This is especially pertinent in chronic diseases such as MPN, where patients may live for decades with ongoing surveillance, repeated laboratory testing, and prolonged exposure to cytoreductive or antithrombotic therapy.

Our results provide an indirect framework for this discussion. AYA patients at NCCCR were not simply a small subgroup within one disease; they represented a substantial proportion of the ET and PV populations and a meaningful minority of MF cases. Even where conventional survival differences were not statistically apparent, these patients still face a long disease trajectory during the most socially and professionally active decades of life. This suggests that age-adapted MPN care should not focus only on thrombosis and transformation, but also on mental health, fertility, adherence, drug toxicity tolerance, and sustainable long-term follow-up. Prospective incorporation of patient-reported outcome tools such as the MPN-SAF TSS would be particularly valuable in future studies from our region.

### Digital Engagement and Patient Education

A further practical consideration for AYA care is how patients seek information and support outside the clinic. Social media communities such as #MPNSM have emerged as disease-specific spaces where patients, clinicians, and advocacy groups exchange information and lived experience.^22^ For a relatively rare disease such as MPN, this can be especially valuable for young patients who may otherwise feel isolated, particularly in multicultural health systems where patients come from diverse educational and linguistic backgrounds. In Qatar, where a large proportion of the population is expatriate and digitally connected, this mode of engagement may be especially relevant.

At the same time, increased digital engagement creates a parallel responsibility for clinicians and centers to provide reliable, comprehensible education. For our patient population, this may mean supplementing routine visits with structured written information on thrombosis risk, pregnancy, fertility preservation, treatment expectations, and warning symptoms that should prompt urgent review. Future center-level initiatives could include patient education pathways, culturally tailored counseling materials, and prospective registries that incorporate both clinical outcomes and quality-of-life measures, thereby building a more complete picture of AYA MPN care in the Gulf region.

## Limitations

This study has several limitations. Its retrospective single-center design may have introduced selection bias, incomplete data capture, and variable follow-up. Small subgroup sizes, particularly among AYA patients with MF, limited statistical power, while the relatively young non-AYA cohort may have attenuated age-related differences. Important clinical variables, including cardiovascular risk factors, treatment exposure, pregnancy outcomes, and some molecular and pathological data, were incompletely available, precluding adjusted analyses. In ET, age-specific molecular comparisons should be interpreted descriptively because molecular and clinical denominators differed. Furthermore, survival and clinical outcomes were analyzed descriptively without multivariable adjustment because of limited event numbers and incomplete covariates. Therefore, the findings should be considered exploratory and hypothesis-generating. Nevertheless, the study provides comprehensive age-stratified data across all three classical Philadelphia-negative MPN subtypes from an underrepresented regional population.

## Conclusion

AYA patients with Philadelphia-negative MPNs at NCCCR, Doha, showed age-associated differences compared with older adults, particularly in ET and PV, where lower-risk classification was more frequent in younger patients, largely reflecting age-based risk models. Molecular analysis demonstrated relative CALR enrichment in AYA ET, balanced JAK2/CALR distribution in AYA MF with JAK2 predominance in older MF patients, and JAK2-mutated PV in nearly all patients, including three with JAK2 exon 12 mutations. Exploratory differences in thrombotic phenotype were observed, whereas overall survival was similar between age groups. These findings support age-aware management and highlight the need for larger prospective multicenter studies from the Middle East with comprehensive molecular and clinical characterization.

## Data Availability

All data generated or analyzed during this study are included in this article. Further enquiries can be directed to the corresponding author.
